# Attributes Development for Pharmaceutical Subsidization: A Qualitative Study

**DOI:** 10.22037/ijpr.2019.15507.13136

**Published:** 2020

**Authors:** Gita Afsharmanesh, Gholamhossein Mehralian, Farzad Peiravian

**Affiliations:** *Department of Pharmacoeconomics and Pharma Management, School of Pharmacy, Shahid Beheshti University of Medical Sciences, Tehran, Iran.*

**Keywords:** Discrete choice experiment, Attribute and attribute-level, Qualitative research, Pharmaceutical subsidization

## Abstract

Discrete choice experiments (DCES) as a stated preference method have used increasingly to determine preferences attached to some attributes associated to health. Although, the validity of this type of studies comprehensively depends on the appropriate determination of attributes and attribute-levels for DCES, there is little rigorous evidence regarding which factors or attributes and attribute- levels should be counted for eliciting public preferences in health resource allocation. This paper responds to such question by carefully doing a qualitative study. A qualitative study used semi-structured interviews, which were audio recorded, transcribed and subject to thematic analysis. Sixteen participants had been key informants and decision makers of pharmaceutical and health system. Initially, by conducting a meticulous literature review, an inclusive list of attributes associated with intended policy was identified. Qualitative data for the development of attributes and their levels were collected using 16 key informant interviews and were analyzed by software MAXQDA followed by a focus group discussion (FGD) with 7 people, well-familiar with the notion pharmaceutical policy and Pharmacoeconomics. The 311 codes in four main dimensions were initially identified by conducting interviews. However, for being manageable within a DCE, they were classified and limited to four attributes, including severity of disease without treatment, health gain after treatment, frequency of patients, and cost of treatment per patient. This qualitative study provides enough evidence for designing and doing a precise discrete choice experiment answering the question about public preferences in pharmaceutical subsidization and contributes empirical evidence to the limited methodological literature on attributes development for DCE, specifically within low and middle-income countries.

## Introduction

Similar to a large number of other countries, in Iran, providing the population with affordable and easy access to safe, effective, and high-quality medicine is one of the primary responsibilities of the Ministry of Health (MOH) (1). For this reason and in order to decrease catastrophic expenditure, the government subsidizes a number of vital and life-saving medicines. However, the question the best way of prioritizing the allocation of scarce health resources to improve the efficiency and equity of access to pharmaceutical products remains unanswered (2–4). So far, to find an answer to this question, policy makers in this field have proposed economic evaluation as the best and the most valued approach. In addition, the quality-adjusted life year (QALY)—a health gain metric which considers the quality of life and length of life—has been widely adopted to measure the value of a health-care treatment (5). Nowadays, identifying and considering the preferences of the target population is one of the approaches which are used by policy makers in various domains such as health care. Thus, it might be argued that in order for a policy design and for the delivery of health services to be efficient, policy makers should adapt their goals to their target society’s preferences. This needs a clear understanding of preferences of the population and their judgment about health programs (6). It has been indicated by studies which investigate public opinions that the public desire to see their preferences considered for “non-technical” dimensions of prioritization (3, 7–9). Similarly, Tappenden *et al.* in a study, argued that the objectives of National Institute for Health and Clinical Excellence (NICE) go beyond achieving technical and allocative efficiency. They also asserted that fair and just distribution of healthcare resources is another key principle which needs to be taken into account (10). The main question here is what other factors the public, who contribute to funding health care by paying taxes, are considered vital in resource allocation policies. Another question to be answered is what distributional weights each individual factor should receive when setting priorities. The health economics research literature presents an extensive investigation of these two questions. However, there is a little agreement on the factors or attributes that the general public desire to be considered and the extent these factors should be taken into account in health resource allocation decisions (2).

The recent literature indicates that a large number of studies in this field are rapidly moving toward applying DCEs which are considered as choice-based techniques (2). DCEs validity, similar to other attribute-based experiments, is largely dependent on the researcher’s ability to identify proper attributes and specify the level of each attribute (11). The process of attribute development should be systematic and meticulous in order for the researcher bias to be avoided (12). In response to this problem, a number of different methods have been applied to develop DCE attributes, including literature reviews, existing conceptual and policy relevant outcome measures, theoretical arguments, expert opinion review, professional recommendations, patient surveys, nominal group ranking techniques, and qualitative research methods (12, 13).

 Considering the limitations of deriving attributes from the existing literature, Coast *et al.* argue that as qualitative studies represent the potential beneficiaries’ perspective and experiences, they are the best source to be used to elicit attributes (12). Such reports, however, are rare in the existing literature in both high- and low-income countries. (14, 15). This study sheds light on the importance of implementing DCE in the health economics, their theoretical foundations and applications, and the logic behind it for two main reasons. The first one is that little is known about DCE in developing countries, especially in Iran, and the second one is that there is a very limited application of DCE in internal studies even in the field of health policy.

## Study setting

In Iran, after the Health Sector Reform, nearly 100% of the population use the national basic insurance system to reimburse their drug expenditures and, most of the time, have to pay around 30% of the costs. However, for decreasing catastrophic expenditure due to high-tech medicines, especially for chronic and life-saving diseases, the government subsidizes some of these pharmaceutical products up to 90% or occasionally 100%. Nevertheless, owing to scarce resources, there is not any rigorous evidence whereby prioritizing could be done. Moreover, it sounds that community preferences, as tax-payers and public budget’s owner, must be considered in this decision-making. 

## Method

This study was grounded on the qualitative approach to elicit the relevant attributes and their levels in order to constitute the DCE framework for conducting quantitative study. In DCEs, each potential product (intervention) is described by its characteristics, which are called attributes, and each attribute receives a range of specific dimensions which are referred to as attribute levels (16). In attempt to generate a set of hypothetical choice alternatives, the attributes of the interventions and their assigned attribute levels are usually combined by applying experimental designs (14, 15). The respondents are asked to present their preference in a questionnaire which represents these competing alternatives (13, 18). The attribute-levels indicate the utility which respondents will align to a particular characteristic of an intervention, and so, their choices or preferences (13, 19) . In the next phase, the main steps taken to conduct the first step of the current study are illustrated below.

*Developing Conceptual framework*

One point which is emphasized in the current DCE literature is that credible attributes and attribute-levels for a DCE need to be policy relevant, sensible and significant to the population being studied and in accordance with the random utility theoretical foundation of DCE (13, 18 and 19). What this signifies is that the appropriate conceptual and theoretical explanatory models as well as empirical literature on the policy issue should be utilized in order to identify such attributes and levels. Therefore, an inclusive list of attributes may be generated through a meticulous review of the existing literature on the policy topic, which may be included in a relevant DCE. 

*Literature review*

The research team, to achieve this goal, conducted a meticulous and comprehensive literature review so that the most relevant attributes influencing the end of the current study would be identified and listed. To do so, two levels of terms were developed and searched in Google scholar, PubMed, Scopus, Science Direct, and Pro Quest databases. The first set of terms incorporated preferences elicitation, pharmaceutical production, discrete choice experiment, subsidization, resource allocation, and the second set of terms included Health insurance, attributes and influential factors, health financing, which were combined with the fires set of terms from time to time. The research team focused on those empirical papers and reviews, policy documents, and conceptual frameworks associated with healthcare financing systems and consumer choice behavior which were published in English during a period of sixteen years from 2000 to 2016. The current paper presents the research team’s insight into that part of the existing literature that guided its qualitative study on the most significant attributes.

*Qualitative study for identification of context-specific attributes*

As mentioned earlier, identifying the relevant attributes and their levels were the main purpose of this study through using a qualitative approach. 

*Study population and sampling*

Qualitative data for the development of context-specific attributes and attribute-levels was collected in a period of 4 months in 2016, using 16 key informant interviews followed by a focus group discussion (FGD) with 7 people of health decision makers and experts in pharmaceutical policy and Pharmacoeconomics. Stratified purposive sampling was used in order to achieve all attributes influencing resource allocation from different social and political perspectives; and the overall sample size was determined by expected saturation point.

*Data collection*

An interview guide was developed based on the conceptual attributes listed according to the existing literature. The literature classifies all the significant attributes in prioritization pharmaceutical funding into three different categories or dimensions: beneficiaries’ characteristics, disease characteristics, and pharmaceutical products characteristics (Table 1). Following that, we modified the guide so that it can be utilized to conduct an interview with experts and decision makers. In order to ensure the consistency of the topics which were supposed to discussed in the interview sessions across all respondents, it was necessary to make use of a guide so that the interviewers may use a common and fixed instrument. This interview guide was comprised of a number of open-ended questions which incorporate adequate probes. The guide contained broad questions on: 1) the aim of subsidizing pharmaceutical products by government and discussing how much we could accede them in order to provide gaps and explore influential attributes which must be considered, 2) the product attributes they would value as important when deciding whether or not to subsidize, 3) the most important diseases which should be prioritized for funding protection, and ultimately 4) the characteristics based on which the patients, who are suffering from these diseases, must be prioritized. Each interview lasted for about an hour, and all the sampled respondents consented to and participated in the study. Before data recording took place, the permission was also obtained from all study participants, and it was allowed for the participants not to continue whenever they would want.

*Data analysis*

Two analysts examined, coded, and categ-orized the qualitative transcripts of the study independently; this rigorous step was taken in an attempt to ensure inter-researcher reliability. The computer assisted qualitative data analysis software MAXQDA (version 10) was used to help the authors to analyze the entire material. To do so, the pre-established coding scheme which was developed based on the generated interview guide and the conceptual attributes listed during the literature review stage was applied. In addition to this, it is expected new codes and categories to emerge in the next stages of reading and so allowed a specific place for them. The other analyst scrutinized the same material manually and examined them inductively to let codes and categories emerge as they proceeded through the transcripts. In the next stage, the two analysts compared the outcome of their analysis in order to finalize an individual list of attributes. The discrepancies resulted from the comparison stage made the analysts to go back to the transcript and finalize their decision as to whether an element reflected a real and reliable attribute or not. 

*Focus group discussion (FGD)*

 The purpose of this step was to limit the number of attributes so that they would be manageable within a DCE. This was done through a discussion of the context-specific attributes which were generated through the qualitative analysis, with “informed” people, who were chosen based on their expertise and experience in healthcare decision making process. The discussion was used as a tool to ensure the consistency of the chosen attributes with the theoretical principles of DCE. Having produced a list of the most significant attributes, the research team discussed it with seven experts who were selected because of their experience with pharmaceutical decision making the Iranian context. We took this step so as to make sure the chosen attributes were credible as well as realistic in the Iranian context and were capable of answering decisive unresolved research questions regarding community preferences for pharmaceutical subsidy. FGD were conducted to draw distinct terms for the development of conceptual attributes, providing a description of key concepts in a few distinct attributes, and deriving attribute levels.

It was conducted in Farsi and by the help of two research assistants serving the role of facilitator and note taker. Preceding the discussion, the assistant serving the role of the facilitator fully explained the research concept to the respondents. Excluding the distractions, on average, FGD lasted 2 h and was tape-recorded and transcribed for later analysis. 

## Results

*Literature review and Qualitative analysis results*

Table 1 displays a list of conceptual attributes which guided the interviews. The two analysts reached an agreement on the complete list of all attributes during the initial triangulation. The 311 codes which were identified consisted of: beneficiaries’ characteristics (44 codes), disease characteristics (118 codes), and pharmaceutical products characteristics (113 codes). Two other dimensions were also defined as social and political considerations (25 codes) as well as interference of insurance organizations for advocating the patients against pharmaceutical expenditures (11 codes).

In line with methodological recommend-ations, the number of attributes to include in the DCE is an important issue to balance. On these bases, as shown in Table 2, eight attributes were taken as the most important attributes that could be feasibly included in the pilot elicitations. They include prevention versus cure, prevalence, severity, socioeconomic status, age, effectiveness, cost, and ethical as well as political consideration. To illuminate more the respondents’ views on attributes, some brief explanations and quotations from the qualitative transcripts are also outlined hereunder.

*Effectiveness*

One of the participants said:

*“Effectiveness and cost-effectiveness of a pharmaceutical product is the best indicator, that is, how much health can be provided for the society?”*

*The cost*

The cost of the unit of medicines, especially for chronic, rare, and orphan diseases, which causes catastrophic expenditures and makes the patients to be impoverished, was considered as another attribute. On the other hand, the limitation of budget is the other more important issue that should be account herein. So, it is critical for decision makers to know how they can provide more health as much as welfare. 

 An example has been outlined by the following statement:

“*Subsidies principally are credits which would be allocated to increase welfare, and no difference is to pharmaceutical products, the aim of resource allocation in each context is accessibility with the least cost to the patients.”*

*Socioeconomic status*

Most of the respondents believed that the socioeconomic status of the beneficiaries should be considered in funding decisions and they proposed it with statements such as:

*“The type of the population is more important than the number of patients, socioeconomic class of the beneficiaries should be considered.”*

*Age*

Many different studies have elicited preferences for age. The majority suggest that the public in general favors the young over the elderly. Preferences for age, or ageism, can be based on a number of ethical principles. Utilitarian ageism is based on a principle of maximizing health gains; as younger patients are expected to live longer than the older patients, there is a greater expected value to save a younger patient. Productivity ageism holds that the very young and the very old have less societal value than individuals at ages in between by virtue of their relative contributions to society. A third conception of ageism stems from a perceived moral obligation to save a young life over an older life because they have had fewer life years. This desire to equalize the age at death is known as egalitarian ageism. The respondents in our study have also mentioned some similar points by statements such as:

*“Young men whit severe disease who has more life expectancy and his normal life has been disturbed should be prioritized.”*

*Severity* *of disease*

Severity of disease was the most important attribute after cost which was mentioned however, as it has been pointed in the literature, it can be defined as the likelihood of death or organ failure as a result of disease progression, independent of treatment and it has different features such as quality of life before treatment, duration of treatment, and quality of life after treatment (25).

*“Financial support of life- saving and disabling diseases is morally justifiable.”*

OR 

*“Diseases without a good prognosis or an effect on the quality of life should not be subsidized.”*

*Prevention vs. cure*

Expected utility theory suggests that a gain of 0.5 QALYs should be valued equally to preventing a loss of 0.5 QALYs; thus, society should be indifferent between acute or preventative care. Some studies have found that respondents strongly favored improvements in health over the prevention of declines and it has been suggested that part of the reluctance to prioritize preventive care may refer in the uncertainty around its effect. On the other hand, the distinction between acute and preventive care, though, may be largely arbitrary. For example, do life-saving treatments improve health or prevent death? However, in our study some respondents due to saving of the health system resources and the importance of the healthcare pointed to this attribute as well.

*“Many complicated diseases would be manageable with a series of preventive interventions.”*

*Prevalence* *of diseases*

Rarity or the prevalence of a specific disease in the population is related to the issue of the distribution of benefits to the many or the few. However, because of the small patient populations of rare diseases, the costs of drug development for such disease can be very high, and the cost-effectiveness of such drugs is often much higher than would generally be accepted. Therefore, the issue in terms of societal preferences is whether the relative rarity of a condition should lead to special consideration in terms of priority and acceptable cost-effectiveness. Consequently, it was considered as another influencing factor for prioritizing in resource allocation.

***“*** *We consider two principle for prioritizing in health; frequency of need and unit cost”.*

*Ethical and political consideration*

All respondents unanimously conceded that pharmaceutical subsidizing has been done based on ethical and political consideration till now.

*Focus group results*

More clarifications of attributes and a lengthier discussion of attribute levels happened during the FGDs. Repeating the discussion in FGDs resulted in retaining 4 of the 8 initially selected attributes which were identified in the qualitative material (Table 3). The process of dropping attributes was guided by multiple criteria. During this process, in an attempt to avoid cognitive inter-attribute correlation, the attributes and their levels which were thought to be overlapping with other attributes were dropped (16). In addition, with the intention of avoiding dominance, some attributes (such as age) with definite preference across some certain levels were discarded in the FGD. On the other hand, although age would be an imperative attribute, FGD were not in favor of this issue that age should be considered as an influencing factor in decision making. As the final step, those attributes which were identified as being of secondary significance such as political considerations which were supposed to have a minor role in identifying the target population’s preferences, were also discarded. However, the fixed levels were defined for all discarded attributes as part of the introduction to the choice exercise. 

As socioeconomic status is relating to cost and it can also be considered as a demographic character for more investigation, it was better to be discarded. 

As most of the respondents believe that cost should be considered as the beneficiaries’ out of pocket rather than the saving or expenditure amount for the government, it was defined in three levels, which nearly implies a proportion of the least income that makes somebody to be poor or put to catastrophic expenditures. It is also concluded that it is better to coalesce effectiveness into defining quality of life after treatment due to describing prognosis of disease, and everyone accepted that the quality of life before treatment is an adaptable definition for severity, and for levels’ comprehensibility, a simplistic scale was adjusted for the questionnaire using “severe disease” and “moderate disease” which would be described in detail in the introductory text according to the health state levels of the EQ-5D classification corresponding to the Mobility, Usual Activities, and Pain/discomfort.

Since prevalence of disease is a determinant attribute especially for constraint budget and severity of orphan disease, as well as their high cost; it was concluded in the study by two levels discriminating orphan diseases from others.

## Discussion

By Providing a clear account of the systematic process of identifying attributes and their levels for a DCE to elicit the preference of the public and decision makers regarding pharmaceutical subsidization in Iran, this study hopes to enrich the literature on this concern (14, 15). The study was commenced by a lengthy process of identifying conceptual attributes from the literature. The later stage was to use the output of the first stage to generate a detailed interview guide which was utilized to collect initial qualitative data within the community using a systematic manner. A rigorous two-step analytical process helped the research team identify relevant attributes and attribute levels. The idea of generating the interview guide based on the results of the literature review, which covered both theoretical and applied studies, helped us identify and generate an initial broad list of attributes and attribute levels which all reflected a significant and policy-relevant element for pharmaceutical subsidization. This initial qualitative phase ensured response efficiency in our DCE, which, in turn, enhance the study’s content validity (11, 13). Furthermore, this qualitative step helped us select potentially dominant and perceptually correlated and exclude less tradable as well as less important attributes and levels from the choice sets. The purpose of this was satisfying the credibility criteria of DCE attributes and their levels (12, 13). While the group of attributes selected in the first stage of the study covered almost all the attributes presented in the literature, in the second stage, the research team attempted to limit the number of them to those attributes which were most relevant to decision making. This process was carried out by a rigorous and reasonable approach. Green and Gerard argued that age is primarily a proxy for capacity to benefit, and, for this reason, it may not be to the advantage or against any specific age groups (29). Additionally, a number of studies have found that while there seems to be a strong evidence in support of prioritizing the young, there is not sufficient evidence on excluding the elderly. This exerted vital effects on the framework of the elicitations or the way the questions were asked during the study. Moreover, according to discussions the prioritizing related to age is impossible, therefor age in spite of importance was deleted. In order to reinforce health maximization, the tension between the interpretations of two types of needs should be highlighted. Therefore, the quality of life before the treatment and the quality gain after the treatment were included. The prioritization of distributing drugs in a community is an issue of prevalence; the drugs for low-prevalence diseases are expensive to develop, and the cost-effectiveness of such drugs is higher than the accepted limits. It is argued that this makes it more difficult for the patients with rare diseases to access potentially beneficial drugs; hence, the prevalence of the disease is considered as the last attribute in design of our study. Since this poses a considerable challenge to the patients suffering from rare diseases to access effective drugs, a disease prevalence was considered as the last attribute in the design of this study.

**Table 1:** Summary of the literature review for attributes

Public Preferences		
**Chance of success, Survival, Quality of life, Additional cost**	P.P for allocation resources for pharmaceutical	Witty *et al.* (2008) Australia (3)
**Gender, smoking status, income (or socioeconomic status), whether the individual maintained a healthy lifestyle, career status and total LE.**	The allocation of health gain between hypothetical groups of potential patients.	Norman, Richard (2013) Australia (22)
**Life expectancy with current treatment, life expectancy gain from new treatment, HRQOL with current treatment, and HRQOL** **gain from new treatment.**	To take BOI into consideration in its appraisals of health technology assessments under the new policy of value-based assessment, to replace the current inclusion of the ‘‘end-of-life’’	Rowen, Donna (2016) U.K (23)
**Life expectancy without treatment;** **Quality-of-life without treatment;** **Life expectancy gain from treatment; Quality-of-life gain from treatment;**	To investigate whether health gains should be weighted differently for different groups of patients.	Shah *et.al* (2015) U.K (24)
**frequency of the disease; cost of treating a single patient with the drug; total cost of funding the drug (budget impact); severity of the disease without the treatment; and impact of drug treatment on a patient’s health**	To investigate individual preferences regarding public funding for drugs used to treat rare diseases and common diseases	Mentzakis *et al. *(2011) Canada (25)
Decision Makers Preferences		
Attributes	**Study focus**	**Study/country**
**Cost effectiveness of treatments, severity of the disease, age and social class**	Preferences of physicians in Côte d’Ivoire when selecting reimbursable pharmaceuticals	Diaby *et al*. (2011) Canada (26)
**Main impact of disease before treatment, annual number of patients to be treated, QALYs gained per treated patient, incremental cost per QALY gained, uncertainty in cost effectiveness is thoroughly explored**	Preferences of All Wales Medicines Strategy Group.(AWMSG) appraisal committee and appraisal .subcommittee (New Medicines Group) members (‘appraisal committees’) for specific new medicines adoption criteria	Linley W and Hughes D (2013) UK (27)
**cost-effectiveness, uncertainty, age of the beneficiary; baseline health status prior to receiving, existence of other alternative treatment**	how efficiency and certain equity objectives are currently being weighted	Tappenden *et al. *(2007) (10)
Public *vs.* Decision Makers Preferences		
Attributes	**Study focus**	**Study/country**
**Chance of response success, survival, quality of life (QoL), cost to government per person treated. Uncertainty around the chance of response success (only for decision makers)**	Evaluate the consistency of public and decision maker preferences for the public subsidy of pharmaceuticals	Whitty *et al.* (2011) Australia (28)

**Table 2:** The first set of attributes confirmed by interviews

Definition	Attributes	Dimensions
**Health care or treatment**	Prevention *vs.* cure (24)	Disease
**Number of patient, rarity of disease**	Prevalence (29)	
**Long side effect, treatment period, QoL before and after treatment, LY gain, end of life**	Severity (65)	
**Having dependency, productivity, the poor, ** **Share of the expenditure** **with pharmaceuticals** **on the monthly income**	Socioeconomic status (33)	Beneficiaries
**Young men are preferable for their productivity and egalitarian aspect**	Age (11)	
**Health gain, QALY gain**	Effectiveness (27)	Pharmaceutical
**OOP, Internal production, catastrophic expenditure, cost effectiveness, the cheaper alternative**	Cost (86)	
**NGO’s force, election propagation**	Ethical and political consideration (25)	Others

**Table 3:** Results of FGD

**levels**	**Attributes**
Moderate, Severe	Severity of disease without treatment
Without changing in QoL, Relative health, Full health	Health acquired after treatment
Rare Not rare	Frequency of patients
Less than 2000,000 Rial (US$ 60)^*^ 2000,000-5000,000 Rial (US$ 60-150) More than 5000,000 Rial (>US$ 150)	Cost of treatment per patient for a month

^*^based on mean currency exchange rate in 2017 according to Central bank of Iran

(https://www.cbi.ir/exrates/rates_fa.aspx, access date 04.05.2018).

## Conclusion

This study provides a comprehensive description of the meticulous application of the recently proposed approaches to the attribute levels development, and it is in line with extant literature on DCE attribute development (12). In the first step, a list of conceptual attributes was obtained through a literature review, which had been classified into three different dimensions: beneficiaries’ characteristics, disease characteristics, and pharmaceutical products characteristics. Based on the results of this phase, interviews were conducted, which led to the identification of eight characteristics as the most important features in this study. In order to reach a reasonable and reliable framework for DCES, a group discussion with the experts was arranged both to reduce the number of features and to provide logical levels for them, so that they can answer all aspects of the research question. Finally, based on the challenges for prioritizing pharmaceutical subsidization in Iran, it was concluded that four main attributes should be presented as: 1) Severity of disease before treatment in two severe and moderate levels, 2) Health level as a result of the treatment at three levels is unchanged, relative improvement and complete recovery, 3) Frequency of disease at two rare and non-rare levels, 4) The amount of payment from the patients’ pocket for medicine at three levels. One of the main goals of this study is to offer further empirical guidance about the methodological processes of identifying and developing attributes and attribute levels for DCEs particularly in LMIC countries. Future studies should focus their attention on providing a clear account of the process of developing attributes of DCEs, as such descriptions are expected to provide fruitful grounds for assessing the accuracy of this process in DCEs. Thus, it might be argued that the potential and prospective of DCE for supporting the design and implementing interventions is highly reliant on the reliability of the attributes and attribute levels developed in the experimental designs.

## References

[1] Ghiasi G, Rashidian A, Kebriaeezadeh A, Salamzadeh J (2016). The impact of the sanctions made against Iran on availability to asthma medicines in Tehran. Iran. J. Pharm. Res..

[2] Gu Y, Lancsar E, Ghijben P, Butler JRG, Donaldson C (2015). Attributes and weights in health care priority setting: A systematic review of what counts and to what extent. Soc. Sci. Med..

[3] Whitty JA, Rundle-Thiele SR, Scuffham PA (2008). Insights into public preferences for pharmaceutical funding. Int. J. Pharm. Healthc. Mark..

[4] Mehralian G, Bastani P (2015). Pharmaceutical strategic purchasing: A key to improve access to medicines. Iran. J. Pharm. Res..

[5] Drummond M (2013). Twenty years of using economic evaluations for drug reimbursement decisions: what has been achieved? J. Health Polit. Polic..

[6] Sobhanian S, Ebadi J, Mehrara M, Akhavan Behbahani A (2015). Discrete choice experiment, an efficient approachto ecconomic valuation of benefits in health projects and policies. Majlis and Rahbord.

[7] Ryan M, Scott DA, Reeves C, Bate A, van Teijlingen ER, Russell EM, Napper M, Robb CM (2001). Eliciting public preferences for healthcare: a systematic review of techniques. Health Technol. Assess..

[8] Wiseman V, Mooney G, Berry G, Tang K (2003). Involving the general public in priority setting: experiences from Australia. Soc. Sci. Med..

[9] van Exel J, Baker R, Mason H, Donaldson C, Brouwer W, EuroVaQ Team (2015). Public views on principles for health care priority setting: findings of a European cross-country study using Q methodology. Soc. Sci. Med..

[10] Tappenden P, Brazier J, Ratcliffe J, Chilcott J (2007). Experiment to Explore NICE Decision Making. Pharmacoeconomics.

[11] Mangham LJ, Hanson K, McPake B (2009). How to do (or not to do) Designing a discrete choice experiment for application in a low-income country. Health Policy Plan..

[12] Coast J, Al-Janabi H, Sutton EJ, Horrocks SA, Vosper AJ, Swancutt DR, Flynn TN (2012). Using qualitative methods for attribute development for discrete choice experiments: issues and recommendations. Health Econ..

[13] Kjær T (2005). A review of the discrete choice experiment - with emphasis on its application in health care. Health Economics Papers.

[14] Hiligsmann M, van Durme C, Geusens P, Dellaert BG, Dirksen CD, van der Weijden T, Reginster JY, Boonen A (2013). Nominal group technique to select attributes for discrete choice experiments: an example for drug treatment choice in osteoporosis. Patient Prefer. Adherence.

[15] Coast J, Horrocks S (2007). Developing attributes and levels for discrete choice experiments using qualitative methods. J. Health Serv. Res. Pol..

[16] Louviere JJ, Pihlens D, Carson R (2011). Design of discrete choice experiments: a discussion of issues that matter in future applied research. J. Choice Model..

[17] Reed Johnson F, Lancsar E, Marshall D, Kilambi V, Mühlbacher A, Regier DA, Bresnahan BW, Kanninen B, Bridges JF (2013). Constructing experimental designs for discrete-choice experiments: report of the ISPOR conjoint analysis experimental design good research practices task force. Value Health.

[18] Clark MD, Determann D, Petrou S, Moro D, de Bekker-Grob EW (2014). Discrete choice experiments in health economics: a review of the literature. Pharmacoeconomics.

[19] Abiiro GA, Leppert G, Mbera GB, Robyn PJ, De Allegri M (2014). Developing attributes and attribute-levels for a discrete choice experiment on micro health insurance in rural Malawi. BMC Health Serv. Res..

[20] Kerssens JJ, Groenewegen PP (2005). Consumer preferences in social health insurance. Eur. J. Health Econ..

[21] Islam T, Louviere JJ, Burke PF (2007). Modeling the effects of including/excluding attributes in choice experiments on systematic and random components. Int. J. Res. Mark..

[22] Norman R, Hall J, Street D, Viney R (2013). Efficiency and equity: a stated preference approach. Health Econ..

[23] Rowen D, Brazier J, Mukuria C, Keetharuth A, Hole AR, Tsuchiya A, Whyte S, Shackley P (2016). Eliciting societal preferences for weighting QALYs for burden of illness and end of life. Med. Decis. Making.

[24] Shah KK, Tsuchiya A, Wailoo AJ (2015). Valuing health at the end of life: a stated preference discrete choice experiment. Soc. Sci. Med..

[25] Mentzakis E, Stefanowska P, Hurley J (2011). A discrete choice experiment investigating preferences for funding drugs used to treat orphan diseases: an exploratory study Emmanouil Mentzakis. Health Econ. Policy. Law.

[26] Diaby V, Dié Kakou H, Lachaine J (2011). Eliciting preferences for reimbursed drugs selection criteria in Côte dʼIvoire. Patient.

[27] Linley WG, Hughes DA (2013). Decision-makers’ preferences for approving new medicines in wales: a discrete-choice experiment with assessment of external validity. Pharmacoeconomics.

[28] Scuffham PA, Whitty JA, Taylor M, Saxby RC (2010). Health system choice: a pilot discrete-choice experiment eliciting the preferences of British and Australian citizens. Appl. Health Econ. Health Policy.

[29] Green C, Gerard K (2009). Exploring the social value of health-care interventions: a stated preference discrete choice experiment. Health Econ..

